# Pneumonia and poverty: a prospective population-based study among children in Brazil

**DOI:** 10.1186/1471-2334-11-180

**Published:** 2011-06-22

**Authors:** Lícia KAM Thörn, Ruth Minamisava, Simonne S Nouer, Luiza H Ribeiro, Ana Lucia Andrade

**Affiliations:** 1 Department of Epidemiology, Secretariat of Health of the Municipality of Goiânia, Brazil; 2 School of Nursing, Federal University of Goiás, Brazil; 3 Department of Preventive Medicine, University of Tennessee Health Science Center, Memphis, TN, USA; 4 Samaritano Hospital of Goiânia, Goiânia, Brazil; 5 Department of Community Health, Institute of Tropical Pathology and Public Health, Federal University of Goias, Brazil

## Abstract

**Background:**

Children in developing country suffer the highest burden of pneumonia. However, few studies have evaluated associations between poverty and pneumonia.

**Methods:**

A prospective population-based study on pneumonia was carried out as part of the Latin America Epidemiological Assessment of Pneumococcus (LEAP study). Chest x-rays were obtained for children one to 35 months old with suspected pneumonia presenting to emergency care centers and hospital emergency rooms in Goiania, Brazil. Chest radiographs were evaluated according to WHO guidelines. Clustering of radiologically-confirmed pneumonia were evaluated using a Poisson-based spatial scan statistic. Associations between census socioeconomic indicators and pneumonia incidence rates were analyzed using generalized linear models.

**Results:**

From May, 2007 to May, 2009, chest radiographs were obtained from 11 521 children with clinical pneumonia; 3955 episodes were classified as radiologically-confirmed. Incidence rates were significantly higher in very low income areas (4825.2 per 10^5^) compared to high income areas (1637.3 per 10^5^). Spatial analysis identified clustering of confirmed pneumonia in Western (RR 1.78; p = 0.001) and Southeast (RR 1.46; p = 0.001) regions of the city, and clustering of hospitalized pneumonia in the Western region (RR 1.69; p = 0.001). Lower income households and illiteracy were associated with pneumonia incidence.

**Conclusions:**

In infants the risk of developing pneumonia is inversely associated with the head of household income and with the woman educational level. Areas with deprived socioeconomic conditions had higher incidence of pneumonia and should be targeted for high vaccination coverage.

## Background

Pneumonia causes substantial morbidity and mortality globally, representing 18% of deaths in children less than five years of age. Almost half of the deaths in 2008 were concentrated in five poor countries in Africa and Asia [1]. Studies have suggested a close association among socioeconomic status, malnutrition and infectious diseases, especially pneumonia, leading to a cycle particularly pernicious for vulnerable children. Evidence exists of the bidirectional causal relationship between pneumonia and poor living conditions, the later frequently encountered in crowding settings, housing with inadequate water and sanitation, where children are repeatedly exposed to viral and bacterial infection [2-4]. For instance, children at day-care centers are at higher risk of getting pneumonia [5].

Prevalence of individual risk factors for bacterial pneumonia, including HIV-infection and severe malnutrition, may contribute to higher pneumonia burden in poorer communities and less developed countries [6]. In regions where *Haemophilus influenzae *type b (Hib) vaccination has not been introduced, pneumonia cases are mainly due to Hib and *Streptococcus pneumoniae *[7,8]*. S. pneumoniae *is the most common etiology of severe pneumonia and the leading cause of vaccine-preventable death in children less than five years of age [9] and several authors have investigated the relationship between socioeconomic status and invasive pneumococcal disease [10,11].

In developing countries, most children with suspicious of pneumonia are more likely to have a bacterial etiology [12]. By using the World Health Organization (WHO) defined chest radiograph-confirmed pneumonia (CXR+Pn) endpoint [13], epidemiologic studies provide an estimate of the vaccine-preventable pneumonia burden [14]. The rationale for use CXR+Pn as proxy for bacterial pneumonia is based on isolation of bacteria from lung aspirates and pleural fluid in cases of CXR+Pn with negative blood culture [15].

There is scarce information measuring the strength of the association between poverty and pneumonia. Previously, we analyzed clustering in Goiania, Brazil of community-acquired, CXR+Pn pneumonia in hospitalized children [16]. To account for the substantial burden of pneumonia that does not result in hospitalization, we used data from population-based study for both ambulatory and hospitalized pneumonia in the Latin American Epidemiologic Assessment of Pneumococcus (LEAP) study to evaluate clustering of CXR+Pn episodes and associations with socioeconomic status.

## Methods

### Study area and population

The study was conducted in Goiânia, capital city of the state of Goias in the central-western Brazil. Goiânia is highly urbanized with an area of 739 492 square kilometers that grew rapidly in the 1990s. In 2009, the population was estimated at 1 281 973 inhabitants, with 51 279 children under four years and infant mortality at 13.3 per 1000 live births [17]. Public health care is provided by Brazil's Unified Health System (SUS). In this network are public, private and university hospitals, which guarantee citizens the constitutional right of access to health care. Brazilians are not required to qualify or register for SUS, as any person in the country can receive free medical care at any private hospital under a SUS contract. An estimated 70% of the population in Goiânia uses the public health care [18]. In Goiania pneumonia cases are first seen at the public healthcare centers (emergency departments) and referred to hospitalization whenever necessary. More than 80% of the hospitalized pneumonia cases are admitted in private hospitals which are reimbursed by both, private insurance and SUS, since the public pediatric hospitals are tertiary hospitals. The vaccine against Hib was introduced into the national immunization program in 1999 and coverage since 2000 has been maintained above 95% [17].

In 2009, when this study finished, pneumococcal conjugate and influenza vaccines were not included in the National Immunization Program, which provides all recommended vaccines at public vaccination clinics. The seven-valent pneumococcal conjugate vaccine (PCV7) and seasonal influenza vaccine were available for purchase in private clinics and free of charge by the Brazilian Ministry of Health for children in high-risk groups. Children at high-risk groups includes children with acquired and congenital immunodeficiency, congenital heart disease, cystic fibrosis and other lung chronic disease, kidney disease, children with no spleen or non-functioning spleen and children with diabetes. Approximately 7% and 10% of one year olds in Goiania had received PCV7 in 2007 and 2008, respectively; 88% were immunized at private clinics. In addition, an estimated 7% of children received influenza vaccines in 2007 and 2008; 84% at private clinics [17].

### Population-based study for pneumonia

**Figure 1:**
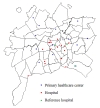
**Geographic location of the participant centers of the population-based surveillance**.

Prospective study for invasive pneumococcal disease (IPD) and pneumonia was conducted between May, 2007 and May, 2009 as part of the LEAP study conducted in Goiânia, Brazil [19,20]. The investigation was conducted at 100% of health services that provide urgent pediatric care in Goiania, which encompasses three public hospitals, 17 private hospitals and 13 public emergency care centers. The map in Figure 1 shows the geographical location of the 33 health facilities involved in the surveillance. Although there are inequalities concerning health care accessibility in several municipalities of the country [4], for Goiania municipality, the government put in action an operational plan to support the choice of the geographical location of the 13 public emergency health facilities for improving health access taking into account the population density and transportation access to the health services [21]. The ultimate purpose was to provide universal and equal access to health services on a decentralized basis.

Children were initially screened by the consultant pediatrician at the participant centers, based on clinician's judgment. Children aged between 28 days to 36 months admitted to emergency rooms and outpatient departments of the healthcare centers or hospitals were eligible if they presented temperature ≥39.0°C and/or suspicion of pneumonia or other invasive pneumococcal disease regardless of temperature. Suspicion of pneumonia followed the guidelines of the WHO which include cough and/or fast breathing [12,22]. IPD is mostly due to meningitis, bacteremic pneumonia, or sepsis and is defined in a patient with a pneumococcal isolate cultured from blood, cerebrospinal fluid, or other normally sterile body fluid.

For the period from May, 2008 to May, 2009 we also enrolled children with temperature ≥39.0°C and flu-like symptoms. A blood sample was collected from each patient on enrollment before antibiotic administration if the clinical situation allowed. Chest x-rays were performed at the health centers at the moment of the enrollment. The decision to refer children to hospitalization based upon the clinician's judgment on the pneumonia severity and on the mother's compliance with antibiotic administration.

The study protocol was approved by national and regional Ethical Review Committees (protocol CONEP # 13 265). A parent or legal guardian provided written consent for patient enrollment. Eligible patients who did not reside in Goiania were excluded.

### Definition of pneumonia and outcomes

#### Primary outcome

CXR+Pn was the primary outcome. CXR+Pn was defined by the presence of alveolar consolidation, pleural effusion or both pattern. Chests radiographs were evaluated by a radiologist trained in reading and interpreting the radiographs according to the WHO guidelines [13], who was blinded to clinical information.

#### Secondary outcome

Hospitalized CXR+Pn cases were considered a proxy for severe pneumonia.

### Socioeconomic variables

The Brazilian Institute of Geography and Statistics defines census tracts as the smallest geographic unit of the municipality for which census and economic data are available. Goiânia is divided in 63 census districts comprising 1066 census tracts designed to be homogeneous with respect to population characteristics, economic status, and living conditions. Based on the proportions of head of households on average less than twice the minimum wage, districts with highest mean income are concentrated near the city center; four areas characterized as very low income are located in the periphery of the city, with newer settlements in the northwest and southwest and more stable communities on the eastern and western sides of Goiania [16].

Seven socioeconomic variables were used to assess risk factors related to radiologically confirmed pneumonia using generalized linear models, described in further section (data analysis). These socioeconomic data for census tracts were obtained from the Brazilian census department [23]. The socioeconomic variables used in this study relied on the census variables collected on 100% of the Brazilian housing units and available by census tracts. Information on race is not collected by the census. A total of seven variables were included in the analysis based on hypothesized relationship between pneumonia and socioeconomic level [24]: (a) percent of head of households earning more than 20 minimum wages monthly. In Brazil, the minimum wage is the lowest amount of salary that employers can legally pay employees for time and efforts spent in producing goods and services (mean of U$ 312.50 per month during the study period); (b) percent of heads of household with more than 15 years of schooling; (c) percent of household with six or more residents; (d) percent of households without bathroom and sewage; (e) percent of households without bathroom but with sewerage; (f) percent of households without piped water supply (water supply was considered inadequate when the water arrives to the dwellings from wells or springs, not channeled into the house); (g) percent of illiterate females greater than 10 years of age.

A socioeconomic score, based on characteristics of the urban districts, was developed to allow assessing the incidence rates of CXR+Pn according to different social classes. The score was also displayed in choropleth map to allow its visual comparison with the spatial patterns of pneumonia incidence within the municipality. Therefore a socioeconomic status rank was created taking into account two census variables: (i) percentage of head of household with more than 20 minimum wages, and (ii) percentage of head of household with more than 15 years of schooling, as a cut-off to have finished college. Initially, the 63 districts were ranked in ascending order by percentage of head of household with more than 20 minimum wages. The lowest socioeconomic status was assigned with the lowest rank, from 1 a maximum of 63. Districts with equal values for this variable were ranked with the same score. The same procedure was applied for the percentage of head of household with more than 15 years of schooling. In a following step, for each district, a socioeconomic indicator was created by adding the two assigned ranks. The created socioeconomic indicator was categorized in quartiles that were named as very low, low, intermediate and high according to their socioeconomic score.

### Geocoding process

CXR+Pn cases were interactively geocoded to the subject's residential address using a digital map dataset (SIGGO v.2 software) provided by the Data Processing Division of the Municipality of Goiânia which displays blocks, areas, lots, streets, and Cartesian coordinates. For analysis purpose, cases were spatially joined to the 63 existing urban districts layer. Therefore, the spatial unit of analysis was the 63 urban districts since each district has homogenous characteristics. A map layer using ArcView^® ^software v.3.2 (Environmental Systems Research Institute, Inc., Redlands, United States) was generated, containing the aggregated number of cases, population and coordinates and area by district.

### Data analysis

Seven socioeconomic variables selected from census were evaluated to assess their association with pneumonia. We used the schooling of women as a predictor for schooling level, instead of schooling of the head of household, because the former are the caregivers of the children. Because frequency of pneumonia cases followed a positively skewed Poisson distribution by district, we evaluated associations between SES variables and pneumonia counts using generalized linear models (GZLM) [25] for negative binomial regression with the logarithm of the population under three years of age included as an offset. All variables were centered to minimize multicollinearity among the predictors.

For spatial analysis purpose, we used the spatial scan statistic [26] (SaTScan^® ^software) to test the null hypothesis that the spatial distribution of pneumonia cases occurs randomly within the municipality. We applied the Poisson model, which relied on aggregated count data to identify clusters of districts with high rates of CXR+Pn. In this methodology a series of windows with elliptical radius ranging from zero to 50% of the total population at risk was established, to ensure that both small and large clusters were found. We used the elliptic spatial scan statistic as it results in better estimate of the true cluster than the circular ones, especially for elongated cluster areas, providing a more specific definition for investigations of cases inside the most likely cluster [27]. The scan statistic used aggregated total number of pneumonia patients and the population at risk in each district and created a large number of elliptical areas (windows) over the study region, each one considered a possible cluster candidate. For each window, the likelihood is calculated based on the observed and expected number of cases inside and outside the area. The area with the maximum likelihood is defined as the most likely cluster. A district is considered to be part of that cluster if its centroid is included on that window. Monte Carlo simulation using 999 replications was used to test for statistical significance. Both most likely cluster and the non-overlapping secondary cluster were reported along with their corresponding relative risks and p values (one tailed). For spatial analysis on CXR+Pn, hospitalized CXR+Pn and for the descriptive analysis we used as denominator the population up to three years of age for each urban district extracted from census [23].

## Results

**Figure 2:**
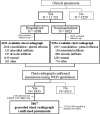
**Flow chart of enrolled subjects and outcome**. Goiânia, Brazil, May/2007-May/2009.

The diagram in Figure 2 outlines the recruitment of cases and CXR+Pn outcomes. Among 14750 children who met inclusion criteria, 11 521 (78.1%) presented with clinical pneumonia and 3229 (21.9%) with other syndromes. Children enrolled with pneumonia for year 1 (May/2007-May/2008) and year 2 (May/2008-May/2009) were 4730 and 6791, respectively. Chest radiographs were obtained for 10 282 (89.2%) cases, being 4328 for hospitalized children (2169/year 1; 2159/year 2). Among the 1239 clinical pneumonia cases without available chest X-ray, almost half were concentrated in northwest Goiania. Pneumonia was classified as CXR+Pn in 3955 (38.5%) of the 10 282 children with clinical pneumonia (1567/year 1; 2388/year 2). Of the 3867 geocoded CXR+Pn cases, 3172 (82.0%) were enrolled at public emergency care centers and 695 (18.0%) at emergency rooms of private hospitals. Among children with available X-ray that was not radiologically confirmed pneumonia, normal radiograph was detected in 3424 children (1420/year 1; 2004/year 2), 285 presented interstitial infiltrate (129/year 1; 156/year 2) and 1337 presented alveolar infiltrate (600/year 1; 737/year 2). A total of 2018 (51.0%) children with CXR+Pn were referred for hospitalization (933/year 1 and 1085/year 2). Hospitalized children with CXR+Pn were statistically younger than those not hospitalized (p = 0.001), although no difference was observed on age of children according to the participant hospitals. Non-hospitalized cases spread more within the municipality area compared with hospitalized children. Hospitalized and non-hospitalized children did not differ by head of household income and schooling. Pneumococci were cultured from blood from 26 (0.7%) children with CXR+Pn.

The median age of CXR+Pn patients was 13 months; 54.0% were male. 18.8% were attending day care center at the time of the enrollment or had attended day care prior to enrollment. Mothers of 0.7% of the case patients were illiterate.

**Figure 3:**
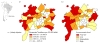
**Pneumonia incidence per 100 000 inhabitants by districts in children aged less than three years old; (a)** Distribution of socioeconomic status by districts **(b)** Goiânia, Brazil, May/2007-May/2009. *Chest radiograph-confirmed pneumonia.

We were able to geocode 3867 (97.8%) of 3955 CXR+Pn cases. Mean annual incidence of CXR+Pn was inversely associated with the socioeconomic ranking of residence's urban district location, ranging from 1637.3 to 4825.2 per 100 000 children, respectively for high and very low income areas (χ^2 ^for trend 161·294; p < 0.001, Table 1). Quartiles of CXR+Pn incidence by districts are shown on choropleth maps (Figure 3a). The higher incidence of CXR+Pn was located in fifteen districts that are dispersed on the periphery of the municipality. Figure 3b highlights 16 districts with lowest socioeconomic conditions, in the same region as districts with high CXR+Pn incidence (Figure 3). Of note is the high incidence of pneumonia in an area with high socioeconomic status located in an extreme northeast district of the city. This district is composed mainly by the Brazilian air force (individuals with high socioeconomic level) and the rest of the district is populated by few poor inhabitants. Despite the overall low usage of pneumococcal conjugate vaccine in Goiânia children who had taken the vaccine were randomly distributed within the municipality (data not shown).

**Table 1:** Incidence of pneumonia confirmed by chest X-ray based on socioeconomic indicator.

Socioeconomic status^a^	Mean annual cases	Population^b^(< 3 years old)	Incidence^c ^per 100 000 (95% CI)
High	125.5	7665	1637.3 (1383.0-1926.0)
Intermediate	339.5	10 976	3093.1 (2790.0-3420.0)
Poor	490.5	14 507	3381.1 (3103.0-3677.0)
Very poor	978.5	20 279	4825.2 (4542.0-5122.0)

Total	1934.0	53 427	3619.9 (3464.0-3781.0)

^a ^The following census/IBGE variables were used to built up the socioeconomic indicator: head of household earning more than 20 minimum wages and with more than 15 years of schooling and head of household income

^b ^Population at risk obtained from census

^c ^Mean annual incidence rate.

Goiânia, Brazil, May/2007-May/2009

**Figure 4:**
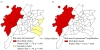
**Risk areas for pneumonia (a) and for pneumonia hospitalization (b) in children aged less than three years old**. Goiânia, Brazil, May/2007-May/2009. *Chest radiograph-confirmed pneumonia.

The spatial scan statistic identified two risk areas for CXR+Pn (Figure 4a). The most likely cluster (RR 1.78; p = 0.001) comprised 15 districts with 1661 observed cases, versus 1150 CXR+Pn expected cases. This cluster included a number of districts in western Goiania. One significant secondary cluster (p = 0.001), composed of three districts, was also identified and was located in the southeast (RR 1.46; p = 0.001). The spatial analysis for hospitalized CXR+Pn patients detected one most likely cluster (RR 1.69; p = 0.001) which overlapped the CXR+Pn risk area (Figure 4b). The majority of pneumonia cases without chest radiography were located in the northwest region, the same region where the most likely cluster of CXR+Pn was located. In this way the relative risk for the spatial scan statistics was conservative but strong enough to show the high risk area in northwest region.

Three out of the seven socioeconomic variables tested in univariate GZLM analysis were statistically associated with pneumonia and therefore were kept in the final multivariate GZLM (Table 2). The percentage of head of households earning more than 20 minimum wages was inversely associated to child pneumonia. For each unit increase in the percentage of head of household earning more than 20 salaries there was a 4.8% decrease in the risk of developing pneumonia. Each unit increase in the percentage of illiterate women (older than 10 years of age) was independently associated with an 11.9% increase in CXR+Pn. Percentage of households without bathrooms was not statistically associated with CXR+Pn in the multivariable model.

**Table 2:** Multivariate generalized linear model applied to socioeconomic variables using number of chest x-ray confirmed pneumonia as dependent variable.

Variables	β	Std. Error	Wald Chi-Square	df	IRR*	95% CI	P value
% of head of households earning > 20 minimum wages	-0.049	0.0080	37.27	1	0.952	0.937-0.967	0.000
% of households without bathroom	0.000	0.0024	0.02	1	1.000	0.995-1.004	0.889
% of woman illiterate > 10 years old	0.113	0.0412	7.48	1	1.119	1.032-1.213	0.006

* IRR - Incidence rate ratio

Goiânia, Brazil, May/2007-May/2009

## Discussion

The findings of this analysis add to ecological studies that have shown high mortality rates of pneumonia in children living in poor regions [6]. In this study the incidence of pneumonia was 80% higher in areas with deprived socioeconomic conditions compared to areas outside the cluster. It is well recognized that increased exposure to viral and bacterial agents in crowded conditions, such as institutionalized environmental, may contribute to increased risk of pneumonia in children [5], besides underlying illnesses as malnutrition [28].

Living conditions and hygiene seem to play an important role in higher risk of pneumonia in low income areas of Goiania. It is not possible to ascertain if poor families seek care later than wealthy families when acute respiratory infection has progressed to pneumonia without early antibiotic treatment. However, evidence exists that the number of people seeking primary health care have markedly increased in recent years and people with serious health disorders are able to seek health care and receive treatment, irrespective of their socioeconomic class [29]. Residences in Goiania are a mean of five km from a pediatric urgent care and transportation is available and affordable.

Previous studies have demonstrated that handwashing is a cost-effective intervention, which can reduce the incidence of community acquired pneumonia by up to 50% [30]. As primary caregivers, mother's education may contribute to compliance with simple prevention and control measures as handwashing. Mothers within the risk areas for pneumonia had lower levels of schooling compared to areas with lower pneumonia incidence. High levels of education can improve access to better job opportunities, feeding practices, and home hygiene [31]. There are many reasons for poverty in the country, and despite governmental initiatives, poverty has remained a serious issue in Brazil. The northwest region of Goiânia, identified as the most likely cluster of pneumonia, was illegally invaded by homeless families at the beginning of the 1980s. Starting in the 1990's the government provided housing for illegal settlers. Even though the northwest area still concentrates poor families and the region is still poorly supplied of sanitation systems and garbage treatment [32]. Four out of the 13 primary health care services are located in that region and 100% of the area is covered by the public family health program. It is worth noting that although families can be illegal settlers, this does not affect their ability to receive public health care in Goiânia municipality.

Studies have shown that surveillance based only on hospitalized pneumonia, underestimates the total burden of pneumonia within the community [33]. In a previous surveillance study on pneumonia in children admitted to hospitals in Goiânia we found an incidence rate of 834.9 per 100 000 in children less than three years of age [16]. The 4.3-fold higher CXR+Pn incidence rate in the present study (3619.0 per 100 000) could be explained by the fact that the inclusion criteria differed between surveillances, the previous one and the present study. The first investigation included hospitalized children with clinical suspicious of pneumonia while the second surveillance enrolled children who presented to both, outpatients or inpatients with flu-like symptoms or clinical suspicious of pneumonia or fever > 39°C. Therefore, the present surveillance used a more sensitive algorithm to capture pneumonia cases, while the previous surveillance captured only inpatient cases, encompassing more severe cases. In this surveillance, 82% of the subjects were enrolled at the outpatient level and only 50.9% of CXR+Pn cases required hospitalization. Therefore, our findings showed that CXR+Pn burden was mainly due to outpatient pneumonia detected at primary care centers.

It is worthwhile mentioning the low rate of use of pneumococcal vaccine by the enrolled children in this study, since the vaccination had not been universally introduced during the study period. Although a considerably number of children had purchased the vaccine in private market, the use of the vaccine did not differ among census tract. A plausible explanation is that the pneumococcal conjugate vaccine is provided free of charge for children at higher risk for pneumococcal diseases by the Brazilian Ministry of Health. Conditions associated to the increased risk of pneumococcal infection include several underlying diseases as detailed in a previous section. The coverage for the PCV7 of 6.6% and 10.5% respectively for 2007 and 2008 during the LEAP study and the high incidence of CXR+Pn points to the need of high and sustainable coverage of pneumococcal vaccination.

Possible reasons for the high incidence of CXR+Pn herein observed could be the dry season with relative little precipitation and about 20% of relative humidity of air during six months of the year. During the dry months, from May to September, the number of visits to ambulatory and hospitalizations due to pneumonia increases. The enrollment of children with flu-like symptoms on the second year of the study also contributed to the high incidence of CXR+Pn since influenza infection may precede bacterial pneumonia [34]. However, pandemic influenza H1N1 was not identified in Brazil until April, 2009 and in Goiânia in June, 2009, one month after the end of this study.

Spatial analysis techniques have been applied to find spatial patterns to identify characteristics of the neighborhood related to the event or to evaluate risk areas for the development of the disease [16,35]. In our study the selection of the spatial scan statistic was based on the aim to locate significant clusters for CXR+Pn with data collected from the entire city. Spatial aggregation provides information about areas, allowing inferences at area level. The use of spatial scan statistic identified priority areas where vaccination coverage should be kept at high rates.

One limitation of this study is the possibility of area-based socioeconomic data may not represent the individual-level socioeconomic status. Using variables from census can lead to a large variability of pneumonia results within the municipality when the spatial unit of analysis is pooled by broad regions. We used the urban districts, which comprise areas similar in socioeconomic characteristics, hence minimizing the variability on the data. Nevertheless our findings should be interpreted with caution due to the possibility of ecological fallacy [36], in which the association between poor areas and pneumonia could be confounded by another factor that actually is leading to pneumonia increase, such as malnutrition, not measured in this investigation. We should also mention that the case definition used in this study based on alveolar consolidation, highly recognized as associated to bacterial pneumonia. It is possible that we missed cases that would have progress from "viral pneumonia pattern" to alveolar consolidation. In addition, most of the missed cases (children without available chest X-ray) resided in the Northeast region. As the high incidence of radiologically pneumonia was also in Northeast region we could assume that potential bias was not directional. It is possible that referral for admission is confounded by socioeconomic status, as a potential source of selection bias. Clinicians might have felt that more reliable and educated families could better manage pneumonia treatment at home. If so, we could expect a selection bias on hospitalization, however without affecting the study outcome. Another concern is how potentially unmeasured socioeconomic factors could have affected the conclusions. For instance, we did not examine the relation between pneumonia and race [37] because race is not available in the Brazilian census. Also, we used hospitalization as a proxy for severe pneumonia, and did not include danger signs such as indrawing of chest wall, cyanosis, inability to feed or drink and hypoxemia to better define severe cases.

The inclusion of pneumococcal conjugate vaccine in the national vaccination programs of various countries has resulted in decreased incidence of pneumococcal pneumonia, all-cause pneumonia, lobar pneumonia, and non-pneumonia acute respiratory infections in children and has protected adults through herd immunity [38,39]. Despite the impact of the vaccination, the vaccine has not been introduced into the routine immunization program of most developing countries; therefore childhood pneumonia remains a major cause of morbidity and mortality in infants of these countries.

Brazil ranks the list of 15 countries with the highest world pneumonia incidence rate [7]. In June, 2010, Brazil introduced a pneumococcal conjugate vaccine containing 10 serotypes into its national immunization program. Studies conducted in the United States identified a decrease in the number of outpatient visits and hospitalizations due to pneumonia for all causes following introduction of pneumococcal conjugate vaccine [38,40]. This study provides a baseline to assess the epidemiological impact of pneumococcal conjugate vaccine on pneumonia incidence in Brazilian children in different socioeconomic strata, in analogy to what we have witnessed in the USA, where a greatest protective effect against IPD was observed in certain ethnic groups such black children compared to the white ones, after vaccination [41]. Since we showed a clear association between pneumonia and poverty, efforts should be made to get poor children adherent to vaccination. The spatial analysis highlighted geographic areas that will need close monitoring for pneumonia.

## Conclusion

In infants the risk of developing pneumonia is inversely associated with the head of household income and with the woman educational level. Areas with deprived socioeconomic conditions had higher incidence of pneumonia and should be targeted for high vaccination coverage.

## Competing interests

In the last 24 months, ALA has received a research grant from Pfizer to conduct the LEAP Study (Latin American Epidemiologic Assessment of Pneumococcus), and served as an advisor/consultant for Pfizer in 2009. As corresponding author, ALA had full access to all the data in the study and had final responsibility for the decision to submit for publication. All other authors declare that they have no conflicts of interest.

## Authors' contributions

ALA conceptualised and designed the study, drafted and revised the report. LKAMT carried out the georeferencing, spatial analysis and epidemiological analysis. LHR performed the chest X-rays reading and interpretation. RM contributed to interpret the data and helped to draft the report, with substantial input to revisions. SSN helped to draft and interpret the spatial statistics and revised the report. All authors were involved in writing the report and approved the final version.

## Funding

Pfizer. ALA (Research Grant no. 306096/2010-2) is a research fellow of the National Council for Scientific and Technological Development (CNPq), Brazil and of the National Institute of Science and Technology for Health Technology Assessment/IATS.

## Pre-publication history

The pre-publication history for this paper can be accessed here:

http://www.biomedcentral.com/1471-2334/11/180/prepub
